# The Gold Coast criteria increases the diagnostic sensitivity for amyotrophic lateral sclerosis in a Chinese population

**DOI:** 10.1186/s40035-021-00253-2

**Published:** 2021-08-09

**Authors:** Dongchao Shen, Xunzhe Yang, Yanying Wang, Di He, Xiaohan Sun, Zhengyi Cai, Jinyue Li, Mingsheng Liu, Liying Cui

**Affiliations:** 1 Department of Neurology, Peking Union Medical College Hospital, Beijing, 100730 China; 2 Neuroscience Center, Chinese Academy of Medical Sciences and Peking Union Medical College, Beijing, 100730 China

**Keywords:** Amyotrophic lateral sclerosis, Diagnostic criteria, Gold Coast, Sensitivity

## Abstract

**Objectives:**

The aim of this study was to assess and compare the diagnostic utility of a new diagnostic criteria for amyotrophic lateral sclerosis (ALS), abbreviated as the ‘Gold Coast Criteria’, with the revised El Escorial (rEEC) and Awaji criteria.

**Methods:**

Clinical and electrophysiological data of 1185 patients from January 2014 to December 2019 in the Peking Union Medical College Hospital ALS database were reviewed. The sensitivity of the Gold Coast criteria was compared to that of the possible rEEC and Awaji criteria (defined by the proportion of patients categorized as definite, probable, or possible ALS).

**Results:**

A final diagnosis of ALS was recorded in 1162 patients. The sensitivity of the Gold Coast criteria (96.6%, 95% confidence interval [CI] = 95.3%–97.5%) was greater than that of the rEEC (85.1%, 95%CI = 82.9%–87.1%) and Awaji (85.3%, 95%CI = 83.2%–87.3%). In addition, the sensitivity of the novel criteria maintained robust across subgroups, and the advantage was more prominent in limb-onset ALS patients and those who completed electromyographic tests. In those who did not achieve any of the rEEC diagnostic categories, the sensitivity of Gold Coast criteria was 84.4%.

**Conclusions:**

The current study demonstrated that the Gold Coast criteria exhibited greater diagnostic sensitivity than the rEEC and Awaji criteria in a Chinese ALS population. The application of the Gold Coast criteria should be considered in clinical practice and future therapeutic trials.

**Supplementary Information:**

The online version contains supplementary material available at 10.1186/s40035-021-00253-2.

## Introduction

Amyotrophic lateral sclerosis (ALS) is the most common form of motor neuron disease (MND), characterized by progressive muscle weakness, atrophy, fasciculations and cramps. The diagnosis of ALS remains phenotypically based, relying on the presence of upper motor neuron (UMN) and lower motor neuron (LMN) signs [1]. Although there is no cure or effective treatment for ALS, an early diagnosis allows recruitment into therapeutic trials and plays an important role in extending the life expectancy for patients.

The revised El Escorial criteria (rEEC) categorize ALS patients into 4 levels of diagnostic certainty, namely clinically definite, probable, laboratory-supported probable and possible ALS [2]. Most randomized control therapeutic trials only recruit definite or probable ALS patients. rEEC has been found with high specificity, but it might delay the diagnosis [3]. The subsequent Awaji criteria propose that the electromyographic (EMG) evidence of abnormalities of LMN is equivalent to clinical examination evidence, and fasciculation potentials are added as LMN signs in addition to neurogenic changes, thus eliminating the clinically probable laboratory-supported ALS category [4]. The Awaji criteria has shown increased sensitivity over rEEC in most studies [5–12], particularly in bulbar-onset ALS patients [12–14]. However, the sensitivity of the Awaji criteria is still not optimal for research purposes, and these two criteria are not intended for clinical practice due to their large complexity and high inter-rater variability even among ALS specialists [15].

To address these weaknesses, a consensus meeting held at Gold Coast in Australia put forward a new set of more simple ALS diagnostic criteria [16]. The diagnostic category in this formulation is a dichotomy, i.e., ALS or not ALS. The Gold Coast criteria for diagnosis of ALS require (1) progressive motor impairment documented by history or repeated clinical assessment, preceded by normal motor function; (2) presence of UMN and LMN signs in at least 1 body region (with UMN and LMN dysfunction noted in the same body region if only one body region is involved) or LMN dysfunction in at least 2 body regions; and (3) investigations excluding other disease processes. In this statement, evidence of LMN involvement can stem from clinical examination and/or from EMG, and evidence of UMN involvement is mainly derived from clinical examination. Findings from ultrasound, transcranial magnetic stimulation, magnetic resonance imaging, and neurofilament levels can be supportive evidence, though the current diagnosis does not require these studies. The appropriate investigations for exclusion of mimicking diseases depend on the clinical presentation, and no minimal or specific suggestions are given in this proposal. It was expected that this new proposal would facilitate early diagnosis and show a better inter-rater agreement while maintaining a high specificity [17].

In this study, we set out to validate the diagnostic utility of the Gold Coast criteria in a clinical ALS database setting, as compared with the rEEC and Awaji criteria.

## Methods

All patients who were suspected as suffering ALS were consecutively enrolled in our ongoing registry platform. Baseline demographic and clinical data were collected using standard spreadsheet during the patient’s first visit to our hospital and during follow-up evaluations, which were performed every 6 months after entering the registry until the patient reached the endpoint (tracheostomy or death) or was lost to follow-up. The clinical diagnosis at the last review was recorded as the final diagnosis. The inclusion time period was from January 2014 to December 2019, which allowed sufficient time for the final diagnosis to be clinically evident. Exclusion criteria included insufficient clinical data to categorize the diagnostic probability and being lost to follow up at 6 months after entering the registry. It should be stressed that patients without EMG examinations in our center were not excluded (only clinical data were used for those who had EMG in the referring hospitals), since the Gold Coast criteria implies that EMG is not required to confirm evidence of LMN dysfunction. However, cases with and without EMG were analyzed separately in subgroup analyses. The details of nerve conduction study (NCS) and EMG recording in our laboratory had been described in previous reports [7, 18].

The clinical notes and results of the NCS and EMG at the first clinical encounter were re-examined by two physicians (DCS and XZY), who were blinded to the final diagnosis at the time of assessment. The clinical/neurophysiological signs were recorded as positive or negative for UMN or LMN in each region of the body. The two physicians graded the patients according to rEEC, Awaji and Gold Coast criteria independently, and disagreements were resolved by consensus.

The diagnoses made with the criteria were then compared with the final diagnoses as a reference standard. In order to calculate the diagnostic sensitivities and specificities, all diagnostic categories in the rEEC and Awaji criteria were determined as having a ‘positive ALS criteria diagnosis’. Conversely, patients who did not achieve a diagnostic category were classified as having a ‘negative ALS criteria diagnosis’. The positive criteria traditionally used in previous studies that only included clinically definite or clinically probable ALS were also tested [5, 14]. The primary outcome measures were the sensitivities and specificities of the three criteria in the diagnosis of ALS. The secondary outcome measures included the diagnostic utility of the criteria in ALS subgroups, divided according to the site of disease onset, or patients with or without EMG. The statistical analyses were performed using SPSS 22.0 (IBM Corporation, Chicago, IL). Data are presented as median (interquartile range) and analyzed using Pearson *χ*^2^ test or McNemar test. *P* < 0.05 was considered as statistically significant.

## Results

**Fig. 1:**
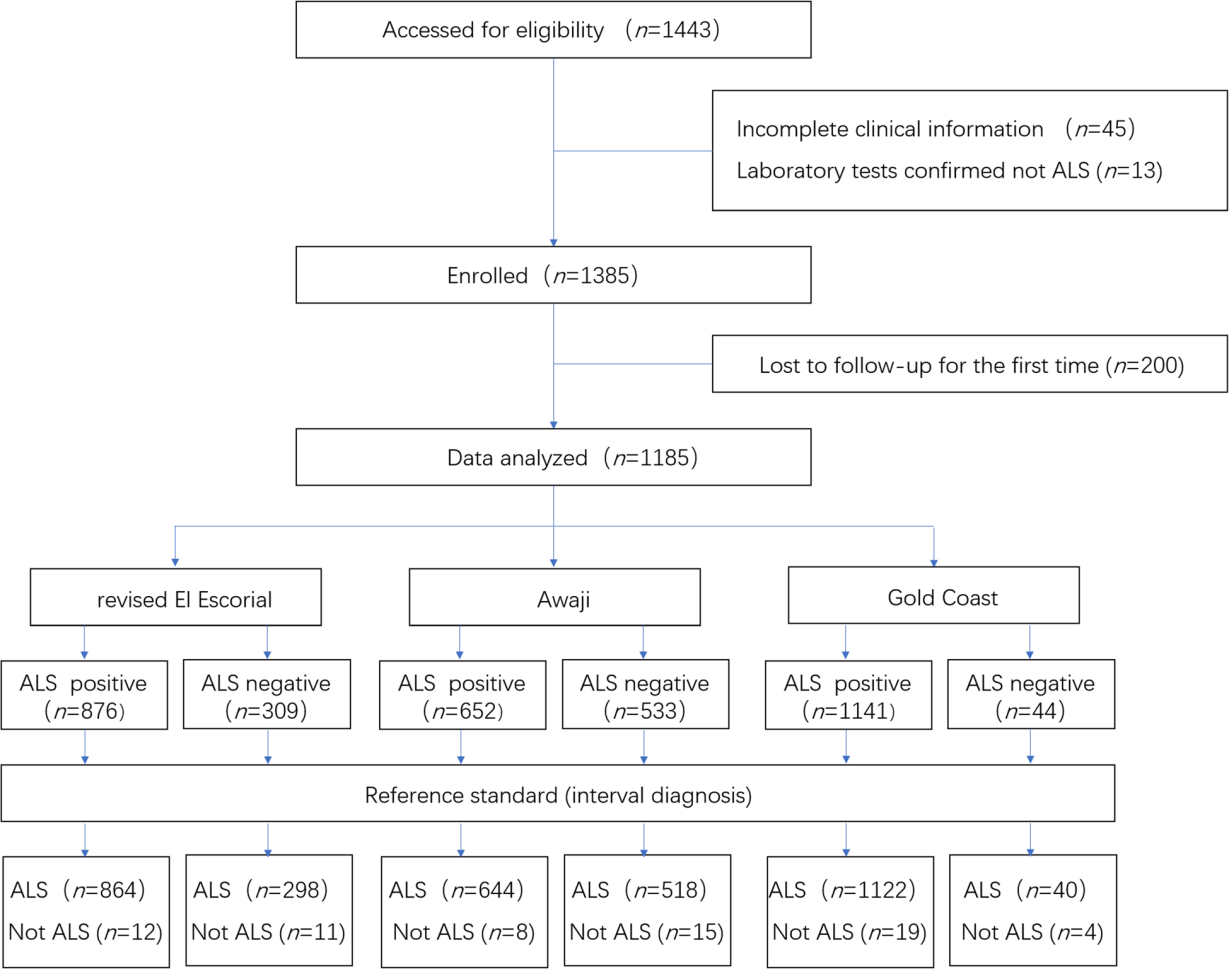
Flow diagram of the study. ALS, amyotrophic lateral sclerosis

During the 6-year duration of the study, 1443 patients entered our registry platform (Fig. 1). Forty-five patients were excluded because they did not see the doctor in person and the clinical information was incomplete. Thirteen cases were diagnosed as non-ALS diseases shortly after registry through laboratory tests, including 1 spinal muscular atrophy, 1 facial onset sensory motor neuronopathy syndrome, 2 Kennedy disease, 4 spinocerebellar ataxias, and 4 showing myogenic damage on EMG. Two hundred patients were lost to follow-up at 6 months after entering the registry. The demographic and clinical details of the included population are summarized in Table 1. Of the 1185 patients who were included in the study, 1162 had a final diagnosis of ALS. During the course of the study, 655 patients with ALS (372 males, 283 females; 441 with EMG, 214 without EMG) reached the endpoint with a median survival time of 31 (22–40) months after disease onset. Twenty-three cases were diagnosed with non-ALS disorders, including 1 immune-mediated polyneuroradiculitis, 1 primary lateral sclerosis, 2 multifocal motor neuropathy (MMN), 2 lumbar spondylosis, 3 cervical spondylosis, and 14 patients who did not have an established diagnosis but all were in stable condition or showed prominent improvement during follow-ups. It should be noted that none of the cases with non-ALS diseases were bulbar-onset. More details of the 23 patients can be found in Supplementary Material.

**Table 1:** Characterization of the study population

	ALS	Non-ALS diseases
*n*	1162	23
Male/Female	632/530	11/12
Onset age (years)	54 (46–61)	44 (37–59)
Disease duration at the first visit (months)	12 (9–21)	24 (10–45)
Bulbar-onset/Limb-onset/Respiratory muscle onset	297/858/7	0/23/0
ALSFRS_R at the first visit	39 (32–43)	43 (38–45)
EMG/without EMG	856/306	20/3

*ALS* amyotrophic lateral sclerosis; *ALSFRS_R* Revised ALS Functional Rating Scale; *EMG* electromyograph

The numbers of included patients in the different diagnostic categories are listed in Table 2 and Table 3 shows the diagnostic accuracy of each type of ALS diagnostic criteria. The diagnostic sensitivities to the definite and probable ALS were 43.6% and 55.4% for rEEC and Awaji, respectively, with a significant difference between them (*P* < 0.001). The specificity of rEEC (69.6%) was significantly higher than that of Awaji (65.2%) (*P* < 0.001). Inclusion of the laboratory-supported probable and possible categories as a positive finding greatly improved the sensitivities of both rEEC (85.1%) and Awaji (85.3%), but the specificities declined sharply (30.4%). As to the Gold Coast criteria, the sensitivity (96.6%, *P* < 0.001) was significantly higher while the specificity (17.4%, *P* = 0.004) being significantly lower than those of the other two criteria.

**Table 2:** Numbers of included patients in the different diagnostic criteria

rEEC	*n*	Awaji				Gold Coast	
		Definite	Probable	Possible	Other	ALS	Not ALS
Total Patients	1185	322	330	356	177	1141	44
Definite	197	197	0	0	0	197	0
Probable	317	82	235	0	0	317	0
Probable LS	362	43	91	222	6	362	0
Possible	129	0	4	125	0	116	13
Other	180	0	0	9	171	149	31
Patients with EMG	876	263	206	271	136	861	15
Definite	138	138	0	0	0	138	0
Probable	193	82	111	0	0	193	0
Probable LS	362	43	91	222	6	362	0
Possible	44	0	4	40	0	39	5
Other	139	0	0	9	130	129	10
Patients without EMG	309	59	124	85	41	280	29
Definite	59	0	0	0	0	59	0
Probable	124	0	124	0	0	124	0
Possible	85	0	0	85	0	77	8
Other	41	0	0	0	41	20	21

*ALS* amyotrophic lateral sclerosis, *EMG* electromyograph, *rEEC* revised El Escorial criteria, *LS* laboratory supported

**Table 3:** Diagnostic accuracy of ALS criteria

	rEEC % (95%CI)	Awaji % (95%CI)	Gold Coast % (95%CI)	*P* values
Total patients				
Sensitivity^a^	43.6 (40.8–46.5)	55.4 (52.5–58.3)		<0.001
Specificity^a^	69.6 (47.0–85.9)	65.2 (42.8–82.8)		<0.001
Sensitivity^b^	85.1 (82.9–87.1)	85.3 (83.2–87.3)	96.6 (95.3–97.5)	<0.001^c^
Specificity^b^	30.4 (14.1–53.0)	30.4 (14.1–53.0)	17.4 (5.7–39.5)	0.004^c^
Bulbar-onset				
Sensitivity^a^	49.8 (44.0–55.7)	56.9 (51.0–62.6)		<0.001
Sensitivity^b^	92.9 (89.2–95.5)	92.9 (89.2–95.5)	96.6 (93.7–98.3)	0.035^c^
Limb-onset				
Sensitivity^a^	41.3 (38.0–44.8)	54.7 (51.3–58.0)		<0.001
Specificity^a^	69.6 (47.0–85.9)	65.2 (42.8–82.8)		<0.001
Sensitivity^b^	82.3 (79.5–84.7)	82.6 (79.9–85.1)	96.5 (95.0–97.6)	<0.001^c^
Specificity^b^	30.4 (14.1–53.0)	30.4 (14.1–53.0)	17.4 (5.7–39.5)	0.004^c^
Patients with EMG				
Sensitivity^a^	38.1 (34.8–41.4)	54.1 (50.7–57.5)		<0.001
Specificity^a^	75.0 (50.6–90.4)	70.0 (45.7–87.2)		<0.001
Sensitivity^b^	84.5 (81.8–86.8)	84.8 (82.2–87.1)	98.6 (97.5–99.2)	<0.001^c^
Specificity^b^	30.0 (12.8–54.3)	30.0 (12.8–54.3)	15.0 (4.0–38.9)	0.018^c^
Patients without EMG				
Sensitivity^a^	59.2 (53.4–64.7)			
Specificity^a^	33.3 (1.8–87.5)			
Sensitivity^b^	86.9 (82.5–90.4)		90.8 (86.9–93.7)	<0.001
Specificity^b^	33.3 (1.8–87.5)		33.3 (1.8–87.5)	0.333

*ALS* amyotrophic lateral sclerosis, *CI* confidential interval, *EMG* electromyograph, *rEEC* revised El Escorial criteria

^a^ Only clinically definite or probable ALS were considered as positive findings

^b^ All diagnostic categories in rEEC and Awaji criteria were considered as positive findings

^c^ Awaji vs Gold Coast criteria

In the limb-onset subgroup (*n* = 881), the diagnostic sensitivity to definite and probable ALS was 54.7% for Awaji, which was significantly higher than 41.3% for rEEC (*P* < 0.001). Similarly, in the bulber-onset group (*n* = 297), the diagnostic sensitivity of Awaji (56.9%) was significantly higher than that of rEEC (49.8%, *P* < 0.001). When considering all diagnostic categories as positive findings, the sensitivities of both rEEC and Awaji were substantially increased, particularly in the bulbar-onset patients, but they were still lower than that of the Gold Coast criteria in both subgroups. The sensitivity gains of the Gold Coast criteria appeared to be more evident in the limb-onset ALS patients, though it showed comparable sensitivities between bulbar- (96.6%) and limb-onset (96.5%) ALS patients. The specificity results remained the same as in the total study population, since all non-ALS disease patients were limb-onset.

When compared to the total study population or patients without EMG, both rEEC (38.1%) and Awaji (54.1%) showed compromised diagnostic sensitivity to definite or probable ALS in the EMG subgroup, but improved diagnostic specificity (75% and 70% respectively). The Gold Coast criteria exhibited the highest sensitivity among the three criteria in both subgroups, and was more sensitive in patients with EMG (98.6%) than in patients without EMG (90.8%) (*P* < 0.001).

There were 180 patients who failed to meet any of the rEEC diagnostic categories, including 155 manifested as LMN syndrome, 19 with UMN signs rostral to LMN signs (9 of whom were classified by Awaji as possible ALS), and 6 isolated bulbar palsy without UMN signs. The sensitivity of Gold Coast criteria in this subgroup was 84.4%.

## Discussion

In total, 1185 suspected ALS patients were enrolled, making this study the largest single-center ALS diagnostic study to date. Results demonstrated a higher sensitivity of the Gold Coast criteria in comparison to the rEEC and Awaji criteria, when considering definite, probable, and possible ALS as a positive diagnosis, and the sensitivity remained robust across subgroups defined by the site of onset, or patients with or without EMG. The sensitivity enhancement of the Gold Coast criteria was more prominent in the limb-onset ALS patients and among those who completed EMG tests. In contrast, the specificity of the Gold Coast criteria was much lower when compared to that of the rEEC and Awaji criteria.

The main limitation of the rEEC and Awaji criteria is the complexity with multiple diagnostic categories. A sizeable proportion of patients do not progress along the diagnostic categories. A fraction of pure LMN-phenotype patients die without fulfilling the criteria [3, 19] while signs of corticospinal tract pathology can be detected in post-mortem studies [20, 21]. A study has found that the diagnostic category of rEEC has no relation with the prognosis [3] and another study proved that only “definite ALS” is a significant factor for poor prognosis [22]. Therefore, the Gold Coast criteria is expected to have higher sensitivity due to the considerable simplification of diagnosis criteria, which particularly classifies LMN dysfunction in at least 2 body regions as a positive sign.

In addition, the present study reaffirmed the higher sensitivity of Awaji in both bulbar- and limb-onset ALS when compared to the rEEC. The inclusion of possible ALS as a positive diagnosis significantly enhanced the sensitivity in both the bulbar- and limb-onset subgroups, with more gains in the bulbar-onset ALS, and the results became comparable between rEEC and Awaji. Bulbar-onset patients are particularly sensitive to Awaji, as bulbar muscles do not show spontaneous activities in the early phase of ALS, but fasciculation potentials and motor unit instability can be easily detected in these muscles [6]. Bulbar involvement is very strong evidence for the diagnosis of ALS in clinical practice, which is supported by the current finding that none of the non-ALS diseases has a bulbar onset. It is obvious that when a patient with bulbar symptoms and progressing limb weakness and wasting that all point exclusively to ALS still cannot meet the criteria of rEEC, a diagnosis of ALS can be made by the Gold Coast criteria. In the present study, the Gold Coast criteria were more sensitive than rEEC and Awaji in both bulbar- and limb-onset patients, particularly in the limb-onset ALS patients.

As far as we know, this is the first diagnostic study of ALS that analyzed patients without EMG, which was an exclusion criterion in previous studies. Of the 309 patients without EMG, 306 were diagnosed as ALS (19.3% definite, 40.5% probable) and 214 reached the end point. The main reasons that ALS patients did not complete EMG in our center included having already received EMG in other hospitals, fulfilling definite or probable categories by clinical data, and intolerance to the tests. According to the new proposal, LMN involvement can be identified by clinical examination and/or EMG, which implies that EMG is not mandatorily required. However, this is certainly not advocated in clinical practice. On the one hand, with clinical examination alone it may be difficult to confirm weakness and wasting in early disease stages, and EMG can detect LMN involvement in non-weak muscles; on the other hand, EMG could provide evidence of progressive neurogenic damage by detecting ongoing denervation. Besides, our subgroup analyses also indicated that EMG can significantly improve the diagnostic sensitivity.

Upadhyay et al. [23] included 156 patients and published the first article on the diagnostic utility of Gold Coast Criteria in MND, in which the sensitivity of revised criteria was 88.2% (135/153) and the diagnostic accuracy was 89%. In a later study of Hannaford et al. including 506 patients, the sensitivity of Gold Coast criteria (92%) was comparable to that of the possible rEEC (88.6%) and Awaji criteria (90.3%) [24]. Specifically, in the latter study, the sensitivity of the Gold Coast criteria was shown to be similar in ALS patients with shorter (< 18 months) and longer (> 18 months) disease durations, as well as in patients with lower (functional scores > 38) and higher severity (functional scores < 38), implying a robust diagnostic utility of the novel criteria throughout the disease process, even in early stages of ALS when functional disability might be still mild [24].

Our result was inconsistent with Hannaford’ study, which showed that the specificity was comparable across the 3 criteria (88.5% for Gold Coast, 96.2% and 95.5% for liberalized rEEC and Awaji). Notably, the specificities of the ALS diagnostic criteria in our study were much lower than those reported previously (all above 80%) [5, 14, 25]. This difference may arise from the different inclusion criteria used. Previous studies assessing the specificity of ALS diagnostic criteria included (1) suspected diagnosis of ALS by the referring physician and neuromuscular disorder, defined as muscle weakness and wasting for at least 6 months in at least 1 body region [14]; or (2) patients who had been referred with MND included in the differential diagnosis [5]. In contrast, our study only included patients highly suspected as ALS, and this is one of the strengths of this study. The ALS diagnostic criteria were designed to be highly specific. Application of full clinical assessments, including laboratory investigations, neuroimaging, muscle biopsy and experimental immunotherapy, to exclude “mimic disorders” is an essential precondition during diagnostic evaluation. We examined the diagnostic criteria in the very population in which the test will be used (those with suspected ALS), and thus provided the most robust estimates of the test’s sensitivity and specificity in clinical settings. In such cases, however, a higher risk of false-positive ALS diagnosis of the Gold Coast criteria would be expected as a result of less comprehensive requirements. A patient with cervical spondylotic myelopathy may fulfil the criteria of disease progression with UMN and LMN signs in one region and an atypical MMN can sometimes fulfil the criteria of progressive LMN damage in two regions. Only longitudinal follow-ups will tell the truth.

Another factor that may contribute to the discrepancies of specificity is the different races of participants. There is evidence that Chinese ALS patients have younger age of disease onset but a longer median survival [26], and more importantly, higher proportions of progressive muscular atrophy (13.1% here compared to 5%–8.9% in previous studies [24, 27]) and other atypical MND in phenotypes [28]. Thus, there are still challenges for differential diagnosis of ALS, particularly the LMN form. The Gold Coast criteria showed a high sensitivity in those who did not achieve a rEEC diagnostic category, highlighting its diagnostic utility in atypical ALS.

It should be noted that 14 patients did not get an established diagnosis, and 2 of them even did not have EMG (No. 11 and 17 in supplement data), but since none of them manifested disease progression, it seems reasonable to determine them as at least not classic ALS. Genetic tests may be beneficial. For example, the SOD1 p.H46R mutation is consistently associated with a relatively benign form of ALS with slow progression [29]. Considering the atypical manifestations and incomplete investigations of these non-ALS diseases in the present study, the reduced specificity of the Gold Coast criteria is less likely to impact patient recruitment in clinical trials. Another limitation of the study lies in the reliability of the clinical and EMG findings because of the retrospective design. The low interobserver reliability of UMN signs may also be a potential source of bias in such studies [15]. Further unbiased, prospective studies are warranted.

## Conclusion

In summary, this hospital-based study indicates that the diagnostic sensitivity to ALS of the Gold Coast criteria is significantly greater than that of rEEC and Awaji in a Chinese population. The novel criteria should be considered as a standard for the diagnosis of ALS in clinical practice, particularly when clinical trials are contemplated.

## Supplementary Information

**Additional file 1: Supplementary file 1.** Demographic and clinical information of patients with non-ALS diseases in this study.

## Data Availability

The datasets used and/or analyzed in the current study are available from the corresponding author on reasonable request.

## Abbreviations

ALS: Amyotrophic lateral sclerosis
EMG: Electromyographic
NCS: Nerve conduction study
LMN: Lower motor neuron
MMN: Multifocal motor neuropathy
MND: Motor neuron disease
rEEC: Revised El Escorial criteria
UMN: Upper motor neuron
