# Supplementary material for: The Gold Coast criteria increases the diagnostic sensitivity for amyotrophic lateral sclerosis in a Chinese population
Source: Transl Neurodegener. 2021 Aug 9;10:28. doi: 10.1186/s40035-021-00253-2 (PMC8351337; doi:10.1186/s40035-021-00253-2)
Supplement: Supplementary file 1 — Additional file 1: Supplementary file 1. Demographic and clinical information of patients with non-ALS diseases in this study. [file 40035_2021_253_MOESM1_ESM.docx]

Demographic and clinical information for patients with non-ALS diseases in this study.

| No. | Sex | Onset age | Disease duration at first visit (months) | Physical Examination | | | EMG damage | First ALSFRS_R | Follow-up (months) | Last ALSFRS_R | rEEC | Awaji | Gold Coast | Interval Diagnosis |
| --- | --- | --- | --- | --- | --- | --- | --- | --- | --- | --- | --- | --- | --- | --- |
|  |  |  |  | Bulbar | Cervical | Lumbosacral |  |  |  |  |  |  |  |  |
| 1 | M | 31 | 24 |  | L | L | B_C_T_LS | 46 | 40 | 47 | Suspected | Suspected | ALS | MMN |
| 2 | F | 37 | 34 |  |  | U_L | L | 46 | 35 | 46 | Possible | Possible | ALS | Lumbar spondylosis |
| 3 | F | 32 | 204 |  | U_L | U_L | No damage | 44 | 31 | 43 | Probable | Probable | ALS | PLS |
| 4 | M | 25 | 6 |  | L |  | C | 48 | 49 | 48 | Suspected | Suspected | not ALS | Cervical spondylosis |
| 5 | M | 37 | 55 |  | L |  | C | 45 | 34 | 47 | Suspected | Suspected | not ALS | Cervical spondylosis |
| 6 | M | 42 | 52 |  |  | L | Not available | 42 | 51 | 45 | Suspected | Suspected | not ALS | Lumbar spondylosis |
| 7 | F | 42 | 13 | U | U_L | U_L | LS | 45 | 67 | 46 | Probable | Probable | ALS | Unknown |
| 8 | M | 66 | 46 |  | L | U_L | B_C_T | 38 | 71 | 42 | Laboratory | Possible | ALS | Unknown |
| 9 | M | 59 | 10 | U_L | U_L | U | B_C_T_LS | 37 | 70 | 48 | Probable | Definite | ALS | Unknown |
| 10 | M | 63 | 10 | L | L | U | C | 36 | 64 | 42 | Possible | Possible | ALS | Unknown |
| 11 | M | 59 | 24 | U_L | U_L | U_L | Not available | 37 | 36 | 42 | Definite | Definite | ALS | Unknown |
| 12 | M | 28 | 7 |  | U_L |  | C_T_LS | 43 | 30 | 46 | Laboratory | Possible | ALS | Immune-mediated polyneuroradiculitis |
| 13 | M | 41 | 43 | U | U_L | U_L | B_C_T_LS | 37 | 23 | 40 | Probable | Definite | ALS | Unknown |
| 14 | M | 44 | 9 | U | U_L | L | C_LS | 42 | 20 | 47 | Laboratory | Possible | ALS | Unknown |
| 15 | F | 39 | 10 |  |  | L | No damage | 37 | 72 | 45 | Suspected | Suspected | not ALS | Unknown |
| 16 | F | 48 | 120 |  | U | L | B_C_T_LS | 45 | 45 | 45 | Laboratory | Possible | ALS | Unknown |
| 17 | F | 53 | 48 | U_L | U_L | U_L | Not available | 42 | 37 | 47 | Definite | Definite | ALS | Unknown |
| 18 | F | 66 | 6 |  | U | U_L | B_C_T_LS | 46 | 29 | 46 | Laboratory | Probable | ALS | Unknown |
| 19 | F | 45 | 26 | U_L | U_L | U_L | B_C_T_LS | 43 | 25 | 44 | Definite | Definite | ALS | Unknown |
| 20 | F | 50 | 3 |  | L | U | B_C | 41 | 15 | 46 | Possible | Possible | ALS | Unknown |
| 21 | F | 60 | 36 |  |  | L | C_T_LS | 46 | 15 | 46 | Suspected | Suspected | ALS | Unknown |
| 22 | F | 46 | 8 |  | U_L | L | C | 37 | 14 | 42 | Possible | Possible | ALS | Cervical spondylosis |
| 23 | F | 43 | 14 |  |  | L | B_C_T_LS | 43 | 12 | 45 | Suspected | Suspected | ALS | MMN |

B: bulbar, C: cervical, L: lower motor neuron involvement, LS: lumbosacral, T: thoracic
